# Awareness and perceptions of child maltreatment among medical students and interns: a cross-sectional study

**DOI:** 10.1186/s12875-025-02729-w

**Published:** 2025-02-14

**Authors:** Sara Owaidah, Reham Alharaz, Sara Aljubran, Zahra Almuhanna, Jawaher AlMusailhi, Turki Abualait, Mona Al-sheikh, Kholoud Al Ghamdi

**Affiliations:** 1 College of Medicine, Imam Abdulrahman Bin Faisal University, Dammam, Saudi Arabia; 2 Department of Psychiatry, College of Medicine, Imam Abdulrahman Bin Faisal University, Dammam, Saudi Arabia; 3 Department of Physical Therapy, College of Applied Medical Sciences, Imam Abdulrahman Bin Faisal University, Dammam, Saudi Arabia; 4 Department of Physiology, College of Medicine, Imam Abdulrahman Bin Faisal University, Dammam, Saudi Arabia

**Keywords:** Child maltreatment, Abuse, Neglect, Sexual abuse, Verbal abuse

## Abstract

Child maltreatment is an issue that burdens communities worldwide. Healthcare professionals, medical staff precisely, play a major role in the reporting responsibility, so it is crucial to ensure that none of the maltreatment cases are missed under medical supervision. This study aims to assess the awareness and perceptions of child maltreatment, and factors that affect reporting these cases among medical students and interns in the Eastern Province of Saudi Arabia. A self-administered questionnaire consisting of five categories (demographic data, the most prevalent form of child maltreatment, awareness and perceptions of child maltreatment, awareness of reporting policy, and factors that contribute to lack of reporting) was distributed among the participants electronically. Data were analyzed using a chi-square test along with frequency distribution of the independent variables. Out of 341 participants, 57.2% were females, and the majority were in their senior years. Less than half of the participants (41.9%) believed that the most common form of child maltreatment is physical abuse. Most participants had adequate awareness of both short- and long-term consequences of child maltreatment. However, they believed that community members did not have adequate awareness about the issue. One-third of the participants believed that they received adequate education on the topic during their training. Furthermore, 78.9% of the participants thought that child maltreatment is often missed. Most participants (90.9%) believed that reporting child maltreatment must be mandatory even if the injuries were not fatal. The top three factors that prevented physicians from reporting child maltreatment were lack of understanding of the reporting procedure, fear of destroying family relationships, and doubt of the occurrence of child maltreatment. Early training and exposure to child maltreatment cases is recommended for all healthcare professionals.

## Introduction

Child maltreatment is a global social problem. Child abuse and neglect (CAN) accounts for 155,000 deaths in children under the age of 15 each year globally, accounting for 0–6% of all recorded deaths [1]. Children who experience any type of CAN during childhood are more likely to be underachievers in school, occupy low-income jobs or become unemployed, experience mental health disorders such as anxiety, depression or PTSD, inflict self-injury or attempt suicide, engage in aggressive risky behavior, or get arrested [1]. An international study demonstrated that 3 out of 4 children between the ages of 2 and 4 years had suffered from psychological and/or physical violence from their parents or caregivers at one point during their lives [2]. Child discipline and parenting techniques in some cultures render the definition of child maltreatment challenging [3]. According to the World Health Organization (WHO) “child maltreatment is when children (< 18 years) have been exposed to any type of abuse or neglect” [2]. Maltreatment has many forms, including neglect and emotional, sexual, and physical abuse. The imprint that CAN leave in a child’s subconscious mind is long-lasting as it debilitates his/her future life in various ways. Future unstable relationships, weak personality, mental disorders, and indulging in risky behaviors are all long-term consequences of CAN [2]. After 1990, the first case of CAN had been reported in Saudi Arabia, which paved the way for a series of CAN reports in the following years [4]. Until the early 2000s, CAN remained unrecognized as a major public health problem. In 2005 the National Family Safety Program (NFSP) was established by the Saudi government, which aided in significant progress in handling CAN cases as they provided a helpline to support a family’s safety as a whole along with training professionals and advocating for a family’s safety and unity [5–6]. Alenezi and colleagues (2021) reported that the prevalence of each type of CAN was as follows: Negligence (39.3%), emotional abuse (32.2%), physical abuse (27.4%), and sexual abuse (19.2%) [7]. A cross-sectional study done in 2012 on 16,939 school children between the ages of 15 and 19 years revealed that the prevalence of each type of CAN in the preceding year was as follows: emotional abuse (65%), negligence (53%), physical abuse (50%), and sexual abuse (10%) [8]. Many CAN cases remain unreported despite the NFSP efforts to establish hospital-based child protection teams, child support hotline, child protection centers (CPC), and to raise awareness of both the medical community and the public [5]. Various studies were done to analyze different factors that prevent healthcare professionals, interns, and medical students from reporting CAN cases [4, 9–11]. The most prominent factors that hamper the reporting process in a healthcare setting were not having enough knowledge to handle such cases, ignorance of reporting protocols, uncertainty of the occurrence of CAN, lack of appropriate training, and averting conflicts with the involved family [4, 9–11]. Most CAN cases are reported in hospital settings, which highlights the critical role of physicians, interns, and students in identifying, detecting, and reporting such occurrences [4]. In 2013 a study was conducted in an Indian nursing school to assess the perception and knowledge of CAN among the nursing students. It revealed that although the knowledge of CAN among students was inadequate, they showed a positive attitude toward the prevention of CAN [12]. Few studies have been carried out to determine the gaps in knowledge and awareness of CAN among Saudi medical students and interns. A cross-sectional study at Al Majmaah University, Saudi Arabia, aimed to assess knowledge, perception, and reporting of child abuse and neglect among interns and medical students [4]. Self-reported questionnaires were distributed among the targeted population over a 9-month period for data collection [4]. Among the 194 participants who were involved in the study, the majority were from fifth year (24.2%), followed by third year (23.2%). The results of the study indicated that 75% of the participants had insufficient knowledge on child abuse and neglect reporting procedures [4]. Approximately 80% of the participants believed that any suspected case of child maltreatment must be reported [4]. Furthermore, more than two-thirds of the participants did not mind attending courses and conferences to improve their knowledge of handling and evaluating cases of child maltreatment [4]. Also, Alkathiri and colleagues (2021) conducted a cross-sectional study to assess the knowledge and attitude toward child abuse among healthcare practitioners in four primary healthcare centers in Riyadh. The study involved 272 health care professionals (primary healthcare physicians, family medicine residents, and nurses). The questionnaire was divided into four sections (demographic data, attitude toward child abuse, the current practice, and evaluation of knowledge). The participants were predominantly females, with a median age of 37.8, and almost 50% were of Saudi nationality. The majority had a vigilant and responsible attitude toward child abuse; more than half of the participants had already handled cases of suspected child abuse during their practice. The findings revealed that while the general knowledge on the subject was excellent, lack of understanding of reporting protocols was a key cause of underreporting [5]. Our study aims to assess the awareness and perceptions of child maltreatment and identify reporting barriers among medical students and interns in the Eastern Province, Saudi Arabia, and hence aid in the establishment of targeted strategies to promote CAN reporting among future healthcare practitioners of this specific population. Results from this study can be compared to outcomes from different regions in Saudi Arabia or global data to provide in-depth insight into this issue.

## Materials and methods

This is a cross-sectional observational study. Data was collected through an online self-reported questionnaire involving **341** adults. The study was conducted from October 2021 to April 2022. The Institutional Review Board (IRB) at Imam Abdulrahman bin Faisal University (IAU), which is in accordance with the Declaration of Helsinki, has approved the study protocol (IRB-Number: IRB-UGS-2021-01-481). The participants included randomly selected males and females who were medical students or interns and who studied in the Eastern Province with a broad representation of nationality and educational level. Before the beginning of the study, informed consent to participate was gathered from all participants. Participants could withdraw from the study at any time as their responses to the questions were optional. A 51-item questionnaire that is divided into five categories was used to assess awareness and perceptions of child maltreatment based on previous studies [4–5]. The first category consisted of seven items to obtain the demographic data (age, sex, university’s name, academic year/internship, marital status, parental status, and nationality). The second category consisted of one item to assess the participants’ awareness of the most common types of CAN in Saudi Arabia. The third category assessed awareness and perceptions of CAN and consisted of 22 items. Yes/No responses were allowed for this section. The fourth category assessed the awareness and perceptions of reporting CAN by using 14 items. Yes/No responses were allowed for this section. The fifth category identified factors that contributed to the lack of reporting, where participants were allowed to choose more than one factor from a seven-item list.

### Data analysis

Data was analyzed by IBM SPSS 26. All categorical variables like sex, age groups, nationality, marital status, participant’s opinion, knowledge, and perceptions were presented as frequency and percentages, while age was presented as mean and standard deviation. The chi-square test was used to compare the proportion between two categorical variables. P-value less than 0.05 was considered significant.

## Results

A total of 341 medical students and interns in the Eastern Province participated in the survey. The study participants were divided into two groups: [1] the junior group, which included third-year medical students and below, and [2] the senior group, which included fourth-year medical students and above, including interns. Out of these, 96 (28.2%) were from the junior group and 245 (71.8%) were from the senior group. The most common academic year was the sixth year (28.2%), followed by the fourth year (22%). The mean (± SD) age of students was 21.8 ± 1.9 years, with most students (76%) being between the ages of 21 and 24 years old. Female participants accounted for 195 (57.2%) of participants, and there was a balanced sex distribution in both groups (*p* = 0.64). Most participants were from IAU (253, 74.2%), while the rest were from King Faisal University (KFU) in Al-Ahsa region. Further details on the demographic data are illustrated in (Table 1).

**Table 1:** Demographic characteristics of participants (*n* = 341)

		Total *n* = 341	Junior students *n* = 96	Senior *n* = 245	*P*-value*
Age (years) Mean ± SD = 21.8 ± 1.9	17–20	71 (20.8%)	66 (68.8%)	5 (2%)	< 0.00
	21–24	259 (76%)	30 (31.3%)	229 (93.5%)	
	> 24	11 (3.2%)	0 (0%)	11 (4.5%)	
Gender	Male	146 (42.8%)	43 (44.8%)	103 (42%)	0.64
	Female	195 (57.2%)	53 (55.2%)	142 (58%)	
University	IAU	253 (74.2%)	76 (79.2%)	177 (72.2%)	0.19
	King Faisal	88 (25.8%)	20 (20.8%)	68 (27.8%)	
Marital Status	Single	313 (91.8%)	93 (96.9%)	220 (89.8%)	0.03
	Married	28 (8.2%)	3 (3.1%)	25 (10.2%)	
Have children	Yes	20 (5.9%)	3 (3.1%)	17 (6.9%)	0.18
	No	321 (94.1%)	93 (96.9%)	228 (93.1%)	
Nationality	Saudi	337 (98.8%)	94 (97.9%)	243 (99.2%)	0.33
	Non-Saudi	4 (1.2%)	2 (2.1%)	2 (0.8%)	

*By Chi-square test, IAU = Imam Abdulrahman Bin Faisal University

**Fig. 1:**
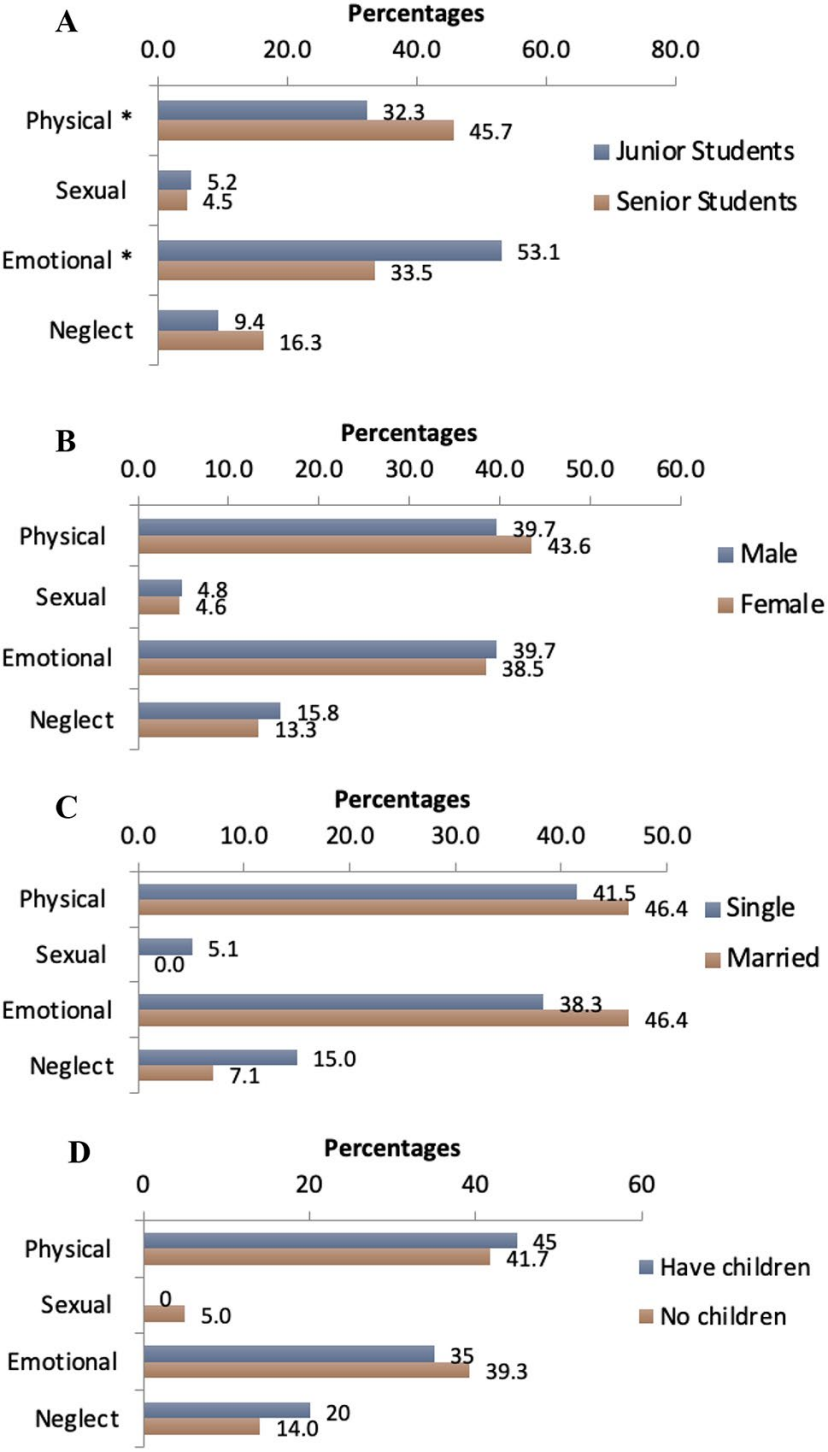
Types of CAN; Participants’ Opinion (*n* = 341) (**A**-Junior Vs Senior Students, **B-** Male Vs Female, **C-** Single Vs married, **D-** Have children Vs No children), *P-value < 0.05

The participants perceived physical abuse as the most common type of abuse, as reported by 143 (41.9%) students, followed by emotional abuse 133 (39%), neglect 49 (14.4%), while sexual abuse ranked in the last place 16 (4.7%). The association between types of CAN and demographic variables (i.e., academic years, sex, marital status, and having children) is presented in Fig. 1. A significant number of senior students (45.7%) perceived physical abuse as the most prevalent type of CAN (*p* = 0.02), while a significant number of junior students (53.1%) perceived emotional abuse as the most prevalent type of CAN (p-value < 0.00). There was no significant difference with regard to sexual abuse and neglect between junior and senior students. This can be attributed to the assumption that junior and senior students perceive both sexual abuse and neglect as equally important.

The assessment of awareness and perception of CAN is presented in Table 2. About 97.7% students thought that CAN exists in Saudi Arabia. Regarding the assessment of CAN’s short- and long-term consequences, more than 90% of the participants indicated that CAN may cause depression in a child’s adult life, lowers a child’s self-esteem, results in fatal injuries, triggers the occurrence of multiple psychological disorders such as schizophrenia, or be a risk factor for becoming an alcoholic. According to 89.1% of students, CAN is underreported. Further details about the perception of CAN revealed that more than 80% believed that the community did not have adequate awareness about the topic (Table 2).

**Table 2:** Awareness and perceptions of CAN (*n* = 341)

Awareness and Perceptions of CAN	Number (percentage)
Child abuse and neglect exists in Saudi Arabia	333 (97.7)
The concept of child abuse and neglect is subjected to the community’s culture and traditions	228 (66.9)
Child abuse and neglect is an Important and sensitive issue in Saudi Arabia	303 (88.9)
Child abuse is underreported in Saudi Arabia	304 (89.1)
Medical learning Institutions provide adequate education on child abuse and neglect	121 (35.5)
Saudi national safety program that deals with suspected cases of child abuse and neglect exists	290 (85.0)
Child abuse and neglect is poorly recognized	269 (78.9)
Child abuse and neglect causes depression in the child’s adult life	335 (98.2)
Child abuse and neglect is a risk factor for becoming an alcoholic in the future	311 (91.2)
Child abuse and neglect is a risk factor of developing serious medical diseases such as cancer in the future	197 (57.8)
Child abuse and neglect triggers the occurrence of multiple psychological disorders such as schizophrenia	315 (92.4)
Child abuse and neglect does not have long term consequences	33 (9.7)
Child abuse and neglect lower a child’s self-esteem	333 (97.7)
Child abuse and neglect may result in fatal injuries	329 (96.5)
I think that parents are allowed to use harsh language while discipline a child	72 (21.1)
I think that parents are allowed to hit their children gently while discipline them as long as it does not result in major physical harms	151 (44.3)
I find it difficult to distinguish between child maltreatment and appropriate discipline	122 (35.8)
I believe Islam approves of scolding kids physically as a method of parenting when a child does not adhere to religious practices such as prayer	225 (66.0)
The community’s knowledge and perception of child abuse and neglect is adequate	52 (15.2)
Health care professionals’ knowledge and perception of child abuse and neglect is adequate	138 (40.5)
I would need advanced training/courses to help me evaluate suspected cases of child abuse and neglect	294 (86.2)
I am willing to take training courses and attend conferences about evaluating and dealing with suspected cases of child abuse and neglect	300 (88.0)

**Fig. 2:**
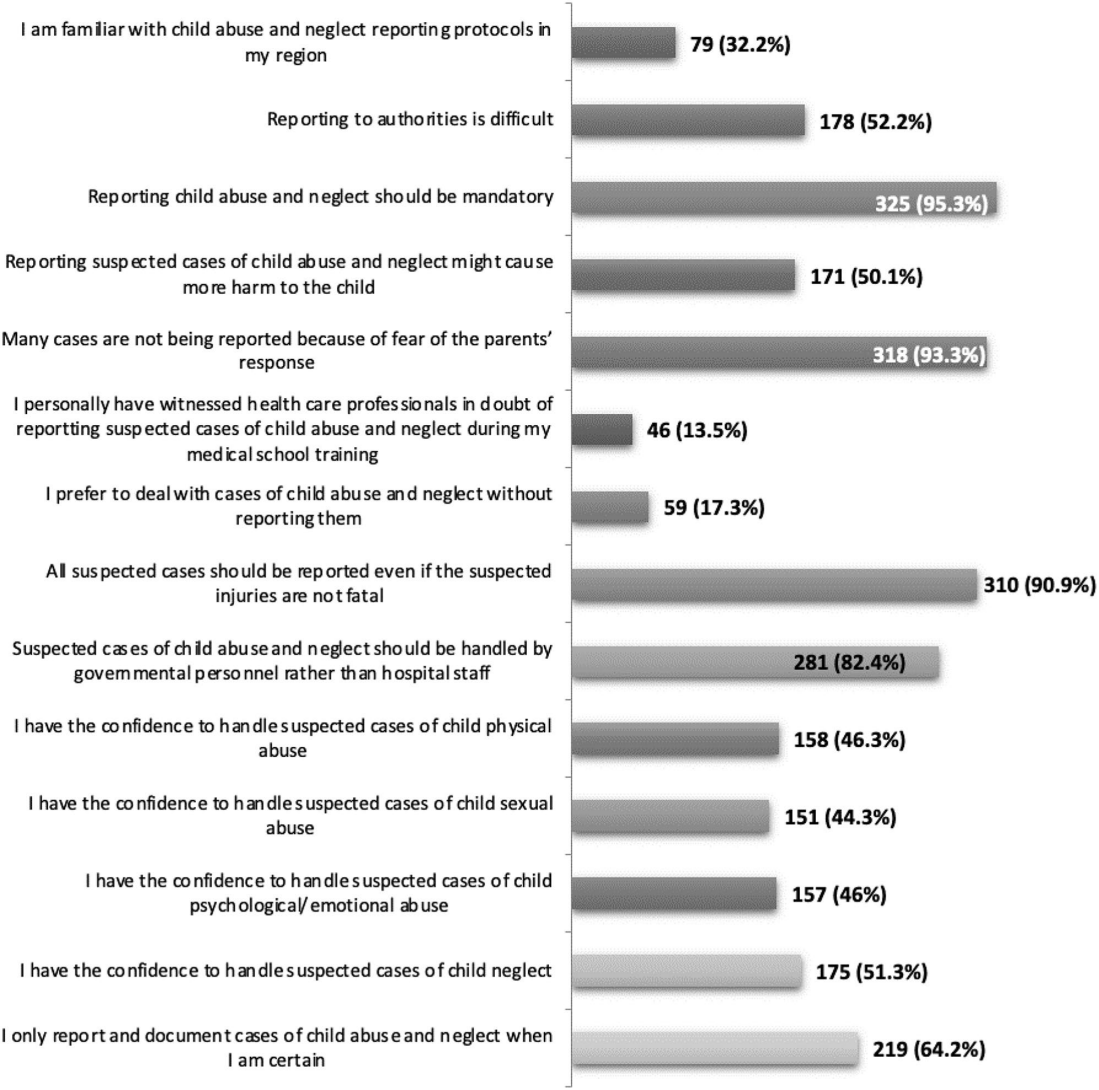
Awareness & Perceptions of Reporting CAN cases (*n* = 341)

**Fig. 3:**
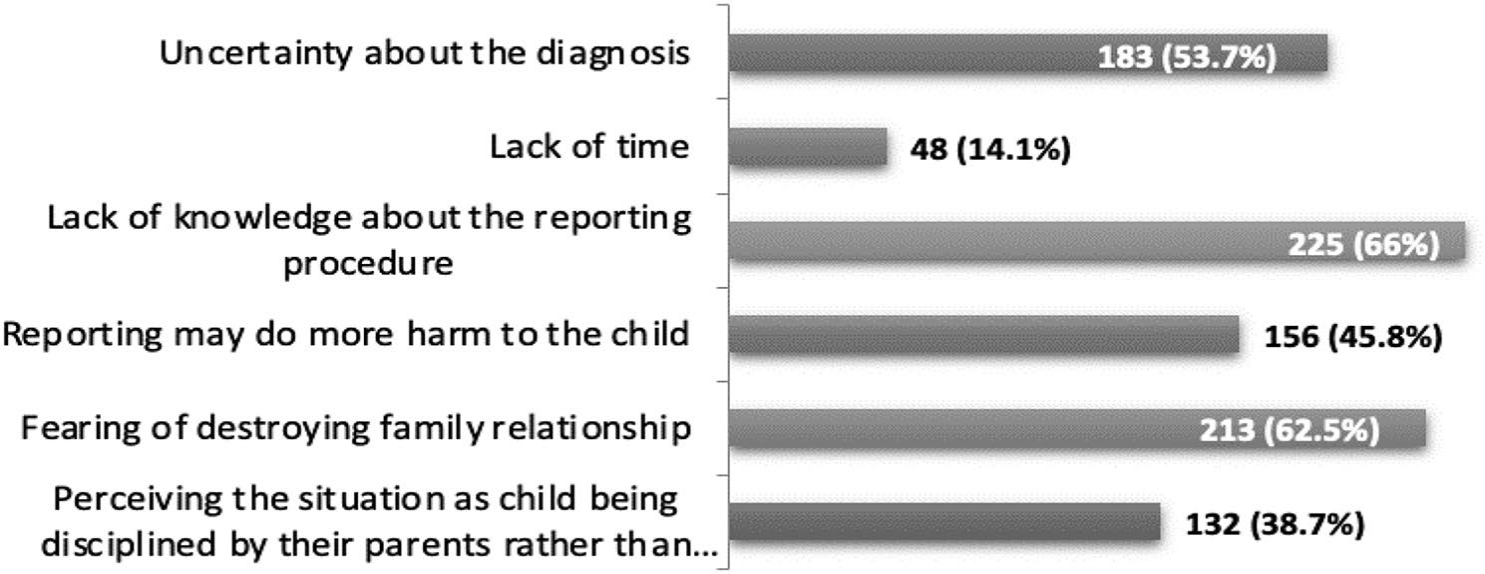
Reasons for not reporting CAN (*n* = 341)

Awareness and perceptions of reporting CAN and reasons for not reporting are presented in Figs. 2 and 3, respectively. Only 32.2% of the participants were familiar with the CAN reporting policy and protocols in their region. Yet, 95.3% believed that reporting suspected CAN is mandatory. The majority (90.9%) suggested that all suspected cases should be reported regardless of the severity of the injury. Most (93.3%) participants believed that many CAN cases are not being reported because of fear of the parent’s response. The four most common causes of not reporting suspecting cases of CAN among the participants were lack of awareness of the reporting procedure (66%), fear of destroying the family relationship (62.5%), uncertainty about the diagnosis (53.7%), and fear that reporting may do more harm to the child (45.8%).

A comparison of knowledge and perceptions of CAN between the academic levels (junior vs. senior) is presented in Table 3. A significantly higher number of senior students considered that CAN is underreported and is an important and sensitive issue in Saudi Arabia as compared to junior students, with p-values of < 0.00 and < 0.01, respectively. Most of the senior students, 220 (89.8%), were aware that the NFSP that deals with suspected cases of CAN exists (p-value < 0.00). All other statements of knowledge and perception about CAN were equally reported by both groups.

**Table 3:** Comparison of knowledge and perception about child abuse and neglect (Junior vs. Senior Students) (*n* = 341)

	Junior students *n* = 96	Senior *n* = 245	*P*-value
Child abuse and neglect exists in Saudi Arabia	92 (95.8%)	241 (98.4%)	0.15
The concept of child abuse and neglect is subjected to the community’s culture and traditions	58 (60.4%)	170 (69.4%)	0.11
*Child abuse and neglect is an Important and sensitive issue in Saudi Arabia*	*78 (81.3%)*	*225 (91.8%)*	*0.01*
*Child abuse is underreported in Saudi Arabia*	*75 (78.1%)*	*229 (93.5%)*	*< 0.00*
Medical learning Institutions provide adequate education on child abuse and neglect	31 (32.3%)	90 (36.7%)	0.44
*Saudi national safety program that deals with suspected cases of child abuse and neglect exists*	*70 (72.9%)*	*220 (89.8%)*	*< 0.00*
Child abuse and neglect is poorly recognized	78 (81.3%)	191 (78%)	0.50
Child abuse and neglect causes depression in the child’s adult life	93 (96.9%)	242 (98.8%)	0.23
Child abuse and neglect is a risk factor for becoming an alcoholic in the future	86 (89.6%)	225 (91.8%)	0.51
Child abuse and neglect is a risk factor of developing serious medical diseases such as cancer in the future	59 (61.5%)	138 (56.3%)	0.39
Child abuse and neglect triggers the occurrence of multiple psychological disorders such as schizophrenia	88 (91.7%)	227 (92.7%)	0.76
Child abuse and neglect does not have long term consequences	8 (8.3%)	25 (10.2%)	0.59
Child abuse and neglect lower a child’s self-esteem	93 (96.9%)	240 (98%)	0.55
Child abuse and neglect may result in fatal injuries	92 (95.8%)	237 (96.7%)	0.68
I think that parents are allowed to use harsh language while discipline a child	26 (27.1%)	46 (18.8%)	0.09
I think that parents are allowed to hit their children gently while discipline them as long as it does not result in major physical harms	49 (51%)	102 (41.6%)	0.12
I find it difficult to distinguish between child maltreatment and appropriate discipline	30 (31.3%)	92 (37.6%)	0.28
I believe Islam approves of scolding kids physically as a method of parenting when a child does not adhere to religious practices such as prayer	68 (70.8%)	157 (64.1%)	0.24
The community’s knowledge and perception of child abuse and neglect is adequate	12 (12.5%)	40 (16.3%)	0.38
Health care professionals’ knowledge and perception of child abuse and neglect is adequate	44 (45.8%)	94 (38.4%)	0.21
*I would need advanced training/courses to help me evaluate suspected cases of child abuse and neglect*	80 (83.3%)	214 (87.3%)	*0.33*
I am willing to take training courses and attend conferences about evaluating and dealing with suspected cases of child abuse and neglect	86 (89.6%)	214 (87.3%)	0.57

A comparison of awareness and perceptions of reporting and reasons for not reporting suspected CAN between the academic levels (junior vs. senior) is presented in Table 4. It revealed that both groups had statistically similar awareness and perceptions of reporting CAN. The only item that received a significantly higher number of responses from junior students was *“Reporting suspected cases of child abuse and neglect might cause more harm to the child”* (p-value < 0.02). All other differences were statistically insignificant.

**Table 4:** Comparison of knowledge and perception about reporting and reasons of not reporting suspected child abuse and neglect cases (Junior vs. Senior Students) (*n* = 341)

Knowledge and perception about reporting	Junior students *n* = 96	Senior *n* = 245	*P*-value
I am familiar with child abuse and neglect reporting protocols in my region	19 (19.8%)	60 (24.5%)	0.35
Reporting to authorities is difficult	54 (56.3%)	124 (50.6%)	0.35
Reporting child abuse and neglect should be mandatory	91 (94.8%)	234 (95.5%)	0.78
Reporting suspected cases of child abuse and neglect might cause more harm to the child	58 (60.4%)	113 (46.1%)	0.02
Many cases are not being reported because of fear of the parents’ response	90 (93.8%)	228 (93.1%)	0.82
I personally have witnessed health care professionals in doubt of reporting suspected cases of child abuse and neglect during my medical school training	12 (12.5%)	34 (13.9%)	0.73
I prefer to deal with cases of child abuse and neglect without reporting them	23 (24%)	36 (14.7%)	0.04
All suspected cases should be reported even if the suspected injuries are not fatal	82 (85.4%)	228 (93.1%)	0.03
Suspected cases of child abuse and neglect should be handled by governmental personnel rather than hospital staff	76 (79.2%)	205 (83.7%)	0.33
I have the confidence to handle suspected cases of child physical abuse	47 (49%)	111 (45.3%)	0.54
I have the confidence to handle suspected cases of child sexual abuse	44 (45.8%)	107 (43.7%)	0.72
I have the confidence to handle suspected cases of child psychological/emotional abuse	48 (50%)	109 (44.5%)	0.36
I have the confidence to handle suspected cases of child neglect	50 (52.1%)	125 (51%)	0.86
I only report and document cases of child abuse and neglect when I am certain	67 (69.8%)	152 (62%)	0.18
Reasons of not reporting			
Uncertainty about the diagnosis	51 (53.1%)	132 (53.9%)	0.90
Lack of time	12 (12.5%)	36 (14.7%)	0.60
Lack of knowledge about the reporting procedure	59 (61.5%)	166 (67.8%)	0.27
Reporting may do more harm to the child	57 (59.4%)	99 (40.4%)	0.00
Fearing of destroying family relationship	63 (65.6%)	150 (61.2%)	0.45
Perceiving the situation as child being disciplined by their parents rather than maltreatment	36 (37.5%)	96 (39.2%)	0.77

A comparison of awareness and perceptions of CAN between both sexes (male vs. female) is presented in Table 5. It revealed that a significantly higher number of male students, 47 (32.2%) and 83 (56.8%), agreed that “*parents are allowed to use harsh language while disciplining a child*” and “*parents are allowed to hit their children gently while disciplining them as long as it does not result in major physical harms*” respectively, as compared to female students (p-value < 0.00). On the contrary, a significantly higher number of female students, 127 (65.1%) and 176 (90.3%), thought “*CAN is a risk factor of developing serious medical diseases such as cancer in the future*” and “*need advanced training/courses to help evaluate suspected cases of CAN” respectively*, as compared to male students (p-value < 0.05). All other statements of awareness and perceptions about CAN were equally perceived by both sexes. A comparison of awareness and perceptions of reporting and reasons for not reporting CAN between both sexes (male vs. female) is presented in Table 3. The following statements “*I personally have witnessed healthcare professionals in doubt of reporting suspected cases of CAN during my medical school training*”, “*I prefer to deal with cases of CAN without reporting them”*, “*I have the confidence to handle suspected cases of child physical abuse*”, “*I have the confidence to handle suspected cases of child sexual abuse*”, “*I have the confidence to handle suspected cases of child psychological/emotional abuse*” and “*I have the confidence to handle suspected cases of child neglect*” were prominent in males (p-value < 0.05). Intriguingly, a significantly higher number of male participants 102 (69.9%) believed that reporting may destroy family relationships as compared to female participants (p-value < 0.02). All other differences were statistically insignificant.

**Table 5:** Comparison of awareness and perceptions of reporting and reasons of not reporting suspected CAN cases (male vs. female) (*n* = 341)

	Male students agreements *n* = 146 (%)	Female students agreements *n* = 195 (%)	*P*-value
I am familiar with child abuse and neglect reporting protocols in my region	40 (27.4)	39 (20)	0.11
Reporting to authorities is difficult	72 (49.3)	106 (54.4)	0.36
Reporting child abuse and neglect should be mandatory	136 (93.2)	189 (96.9)	0.10
Reporting suspected cases of child abuse and neglect might cause more harm to the child	75 (51.4)	96 (49.2)	0.70
Many cases are not being reported because of fear of the parents’ response	140 (95.9)	178 (91.3)	0.09
I personally have witnessed health care professionals in doubt of reporting suspected cases of child abuse and neglect during my medical school training	27 (18.5)	19 (9.7)	0.02
I prefer to deal with cases of child abuse and neglect without reporting them	41 (28.1)	18 (9.2)	< 0.0
All suspected cases should be reported even if the suspected injuries are not fatal	128 (87.7)	182 (93.3)	0.07
Suspected cases of child abuse and neglect should be handled by governmental personnel rather than hospital staff	114 (78.1)	167 (85.6)	0.07
I have the confidence to handle suspected cases of child physical abuse	79 (54.1)	79 (40.5)	0.01
I have the confidence to handle suspected cases of child sexual abuse	77 (52.7)	74 (37.9)	0.01
I have the confidence to handle suspected cases of child psychological/emotional abuse	81 (55.5)	76 (39)	0.00
I have the confidence to handle suspected cases of child neglect	85 (58.2)	90 (46.2)	0.03
I only report and document cases of child abuse and neglect when I am certain	101 (69.2)	118 (60.5)	0.09
**Reasons of not reporting suspected CAN cases**			
Uncertainty about the diagnosis	70 (47.9)	113 (57.9)	0.07
Lack of time	25 (17.1)	23 (11.8)	0.16
Lack of knowledge about the reporting procedure	95 (65.1)	130 (66.7)	0.76
Reporting may do more harm to the child	68 (46.6)	88 (45.1)	0.79
Fearing of destroying family relationship	102 (69.9)	111 (56.9)	0.02
Perceiving the situation as child being disciplined by their parents rather than maltreatment	56 (38.4)	76 (39)	0.91

Comparison of awareness and perceptions of CAN between single and married participants revealed that both groups had similar awareness and perceptions of CAN. The only item that revealed a significantly higher agreement in single students (p-value = 0.02) was “*I have the confidence to handle suspected cases of child psychological/emotional abuse*”.

## Discussion

Current findings of this study showed that physical abuse was perceived as the most frequently reported abuse by medical students and interns, which is consistent with findings of previous studies [4]. In contrast, Alnasser and colleagues (2017) have reported that sexual abuse was acknowledged as the most common type of reported child maltreatment [13]. Such discrepancies between these findings might be attributed to cultural variations between the two regions in which these studies took place. The NFSP acknowledged that neglect is the most common type of reported abuse, followed by physical abuse [7]. Child neglect and physical abuse interconnect with one another as both being manifested in physical injuries, which may be misleading in determining the type of abuse without a thorough history. Medical students and interns are rarely involved with the progression of the reported case; they may miss the final reported type of CAN and attribute the physical injuries to the most obvious type according to what they witnessed, physical abuse. In line with previous studies, the majority of participants were aware of the existence of CAN in Saudi Arabia and that it is underreported [3–4]. A positive attitude toward the willingness to attend courses to acquire new skills in handling CAN cases was found in our study as well as most studies on healthcare practitioners and students [4–5, 9, 13]. This may be attributed to the awareness of educated members of the society about the importance of knowledge acquisition in this topic. The basic knowledge about short- and long-term consequences of CAN was adequate in most of the participants in this study as well as in Alanazi study [4]. Also, a study in Riyadh reported that the participants believe they have received adequate education about CAN during their training [14], while this study reported that only one-third of the participants believed so. The mismatch between studies can be attributed to the difference in the cultural background of the different regions where the 2 studies took place and different educational institutions [4, 14]. Only one-third of the participants were aware of reporting protocols in their region, which explains why despite adequate knowledge and the willingness to report, many CAN cases remain undetected. The most common barriers to reporting suspected CAN cases were lack of knowledge on how to report, fear of ruining family relationships, and uncertainty about the diagnosis, which were similar among different studies, yet with varying percentages [3, 4, 9, 14]. This study compared junior to senior participants responses and found a significantly higher agreement by seniors that CAN is underreported and is an important and sensitive issue in Saudi Arabia compared to junior students. Moreover, the majority of seniors were aware that the NFSP exists in comparison to juniors. One possible explanation for this is that seniors have more practical training and are exposed to cases. Another study showed that senior students were more aware of the existence of CAN, and they were more eager to participate in training courses [4]. Regarding awareness and perceptions about reporting CAN cases, a higher number of responses from the junior group believed that reporting may inflict more harm upon the child compared with the senior group. However, other studies showed no significant difference between junior and senior students with regard to this issue [4]. A study that created a model connecting the actions and results of medical students learning in the workplace noted that juniors in their first years of medical school are reluctant to participate clinically in various scenarios as they are yet to develop a sense of confidence in their skills compared with their senior peers [15]. A significantly higher number of male participants agreed that using harsh language and gentle hitting were acceptable parenting methods. This might be due to a cultural background that promotes masculinity as a virtue rather than a vice. Men are socially conditioned to be stronger and more authoritative than women [16]. A study conducted in the United States of America demonstrated that men lean toward harsh discipline. Fathers in the study were more likely to practice dysfunctional parenting approaches rather than taking the time to have purposeful conversations with their children [17]. The current study showed that the top three factors that prevented physicians from reporting child maltreatment were lack of understanding of the reporting procedure, fear of destroying family relationships, and doubt of the occurrence of child maltreatment.

## Conclusion

This study showed that there is adequate awareness and perceptions of child maltreatment among medical students and interns in the Eastern Province of Saudi Arabia. Barriers to reporting were due to a variety of reasons that can all be tackled by training and education from the point of view of medical students and interns. We recommend that medical curricula address outcome-based specific training on how to detect and report cases of CAN, reporting channels, and protocols. This study is a call for community service activists and social workers to raise awareness regarding the importance of observing children and reporting any form of abuse to the authorities, which entails providing easily accessible reporting channels and establishing an integrated system that ensures applying deserved penalties to perpetrators along with ensuring full protection and safety to victims. This should not only prevent abusive behaviors but also prevent the long-term effects of abuse on the candidates themselves and on the community and assure a better quality of life for all.

### Limitations and recommendations

A major strength of this study is that it assessed the influence of cultural factors, including religious beliefs and discipline methods, on the perception of CAN, which narrowed the gap in the understanding of deep-rooted issues interfering with the elimination of child maltreatment. However, some limitations should be considered in interpretations of the current results. This study relied solely on self-reported questionnaires. Face-to-face interviews might have revealed different and/or in-depth findings. Participants are more likely to select the most ideal response rather than being truthful. To address this problem, observational studies are suggested. Furthermore, because this study is limited to a single region in Saudi Arabia, further research on the topic is encouraged in other regions in Saudi Arabia, as well as the inclusion of other healthcare specialties in the study, to obtain a better overview. According to our study, the foundation of the lack of reporting was uncertainty regarding how to report. Doubting the purpose of doing good or even trust in one’s judgment. For that, establishing policies that assure appropriate incident reporting, investigation, assigning a just penalty, and assuring a better quality of life for the child is crucial, as healthcare practitioners’ role is to report suspicion, not to confirm the occurrence of the incident nor to assign judgments on offenders or provide safe homes for victims of CAN.

## Data Availability

Data are available on reasonable request from the coresponding author.

## References

[1] Gilbert R, Widom CS, Browne K, Fergusson D, Webb E, Janson S. Burden and consequences of child maltreatment in high-income countries. Lancet. 2009;373(9657):68–81. 19056114 10.1016/S0140-6736(08)61706-7

[2] World Health Organization. Child maltreatment. https://www.who.int/news-room/fact-sheets/detail/child-maltreatment (2020, accessed 25 Feb 2022).

[3] Aldukhayel A, Aljarbou E, Alturki F, et al. Knowledge and attitude regarding child abuse among primary Healthcare Physicians and interns in Al Qassim, Saudi Arabia. Cureus J Med Sci. 2020;12(12):e12270. 10.7759/cureus.12270 PMC7773291 33403187

[4] Alanazi S, Althaqib A, Albeladi K, et al. Child abuse and neglect awareness between knowledge, perception, and reporting among interns and medical students of Majmaah University. IJMDC. 2021;5(2):607–13.

[5] Alkathiri M, Baraja M, Alaqeel S. Knowledge, attitude, and practice regarding child maltreatment among health care providers working in primary care centers in Riyadh, Saudi Arabia. J Family Med Prim Care. 2021;10(9):3198. 34760730 10.4103/jfmpc.jfmpc_2492_20 PMC8565144

[6] The National Family Safety Program Summary. The National Family Safety Program. [accessed 21 Oct 2024] Available from: https://httpwwwwforsaudiwomencom.weebly.com/uploads/1/9/3/6/19369117/_english_nfsp_summary-2.pdf

[7] Alenezi S, Alnamnakani M, Murshid R, et al. Evaluation of the impact of the COVID-19 pandemic on the reporting of maltreatment cases to the National Family Safety Program in Saudi Arabia. Child Abuse Negl. 2021;122:105297. 34481139 10.1016/j.chiabu.2021.105297 PMC8514881

[8] Al-Eissa M, Saleheen H, AlMadani S, et al. Determining prevalence of maltreatment among children in the Kingdom of Saudi Arabia. Child Care Health Dev. 2016;42(4):565–71. 26879326 10.1111/cch.12325

[9] Alshouimi OA, Almusaad MN, Aldawood MM, et al. Medical students’ knowledge of child abuse and neglect in Saudi Arabia: a cross-sectional study. Med Sci. 2021;25(114):1844–50.

[10] Elarousy W, Abed S. Barriers that inhibit reporting suspected cases of child abuse and neglect among nurses in a public hospital, Jeddah, Saudi Arabia. East Mediterr Health J. 2019;25(06):413–21. 31469161 10.26719/emhj.18.055

[11] Alrabah B, Horaib Y, Alharbi M, et al. Child abuse and Pediatrician’s role in Riyadh, Saudi Arabia. Int J Med Res Prof. 2018;4(1):151–56.

[12] Poreddi V, Pashapu D, Kathyayani B, et al. Nursing students’ knowledge of child abuse and neglect in India. Br J Nurs. 2016;25(5):264–8. 26972999 10.12968/bjon.2016.25.5.264

[13] Alnasser Y, Albijadi A, Abdullahb W, et al. Child maltreatment between knowledge, attitude and beliefs among Saudi pediatricians, pediatric residency trainees and medical students. Ann Med Surg. 2017;16(2):7–13. 10.1016/j.amsu.2017.02.008 PMC5329067 28275426

[14] Gopalakrishna V, Basheer B, Alzomaili A, et al. Knowledge and attitudes toward child abuse and neglect among medical and dental undergraduate students and interns in Riyadh, Saudi Arabia. Imam J Appl Sci. 2020;5:38–46.

[15] Dornan T, Boshuizen H, King N, Scherpbier A. Experience-based learning: a model linking the processes and outcomes of medical students’ workplace learning. Med Educ. 2007;41(1):84–91. 17209896 10.1111/j.1365-2929.2006.02652.x

[16] Nahshal MM. Masculinity in Saudi Arabia Where we are and where we go from here. Int J Hum Soc. 2019;9(1):115–25.

[17] Vally Z, Hichami FE. Knowledge about parenting as a predictor of behavioral Discipline practices between mothers and fathers. Psychol Stud. 2020;65(1):40–50.

